# Ambient Processed, Water-Stable, Aqueous-Gated sub 1 V n-type Carbon Nanotube Field Effect Transistor

**DOI:** 10.1038/s41598-018-29882-w

**Published:** 2018-07-30

**Authors:** Saumya Joshi, Vijay Deep Bhatt, Ewa Jaworska, Agata Michalska, Krzysztof Maksymiuk, Markus Becherer, Alessio Gagliardi, Paolo Lugli

**Affiliations:** 1 Department of Electrical and Computer Engineering, Technische Universität München, Munich, 80333 Germany; 2 Faculty of Chemistry, University of Warsaw, 02093 Warsaw, Poland; 3 Faculty of Science and Technology, Free University of Bozen-Bolzano, 39100 Bolzano, Italy

## Abstract

In this paper we report for the first time an n-type carbon nanotube field effect transistor which is air- and water-stable, a necessary requirement for electrolyte gated CMOS circuit operation. The device is obtained through a simple process, where the native p-type transistor is converted to an n-type. This conversion is achieved by applying a tailor composed lipophilic membrane containing ion exchanger on the active channel area of the transistor. To demonstrate the use of this transistor in sensing applications, a pH sensor is fabricated. An electrolyte gated CMOS inverter using the herein proposed novel n-type transistor and a classical p-type transistor is demonstrated.

## Introduction

Over the past years carbon nanotubes (CNTs) have gathered significant attraction attributed to their unique chemical, mechanical and electronic properties^1^. In addition to the extraordinary electrical properties of carbon nanotubes, the ability to fabricate solution processable field effect transistors by low temperature processes has made them very appealing candidate in flexible electronics. Carbon nanotube field effect transistors (CNTFETs) which are gated by an electrolyte, are highly sought candidates for chemical- and bio- sensing applications. CNTs exhibit either metallic or semiconducting behavior, depending on their chirality^2^. When semiconducting CNTs are used as active element in the field-effect transistors (FETs)^3^, the as fabricated FETs behave as p-type due to the electron withdrawal effects by the adsorbed oxygen on nanotube surface^4^, hence an absence of electron current^5^. However, the capability to fabricate n-type CNTFETs along with the p-type is of high technological relevance, as this permits fabrication of complementary metal oxide semiconductor (CMOS) circuits that use both the p-type and n-type SWNTs^6^. The fabricated CMOS circuits offer several advantages including higher noise margin, lower power consumption, simpler circuit design and higher circuit yield^7,8^. Nevertheless, fabrication of n-type CNTFET preferably in a relatively simple process, and at the same time operation in aqueous environment has been a real challenge.

Successful attempts have been made in the past to produce n-type CNTFETs involving removal of adsorbed oxygen by annealing in vacuum^9^ or an inert gas^10^ environment, even though n-type CNTFETs fabricated by such annealing process are reversible and on exposure to oxygen again turn to p-type. Towards finding a better solution, n-type CNTFETs were fabricated by n-doping the CNTs with electron donor materials^10,11^ and were further often protected by a passivation layer^5^. Compounds like poly(ethyleneimine) (PEI)^12^, benzyl viologens (BV)^13,14^, dimethyl-dihydro-benzoimi-dazoles (DMBI)^6^, dihydronicotinamide adenine dinucleotide (NADH)^15^, hydrazine^16^, decamethylcobaltocene^17^ have also been demonstrated in recent past as effective solution processable materials with the ability to convert p-type FETs to n-type after it is deposited on the surface of CNTs. Though these modified CNTFETs are air-stable, so far none of these systems have been demonstrated to be water stable such that the modified n-type CNTFETs can be used in aqueous-electrolyte gated configurations.

The ability to use the device in aqueous solutions opens the possibility of using electrolyte-gated(EG) FETs for bio-chemical detection applications^18^. The main motivation to use EGFETs is their high capacitance value (μF/cm^2^) that exceeds any conventional high-K dielectric or any ultra-thin self assembled monolayer (SAM) serving as dielectric^19^. Other important characteristics of EGFETs that are of interest include low source and drain contact resistances^20^, ease of integrating EGFETs as bio-chemical sensors^21^, capability of solution processable solid dielectrics^22^, low operational voltage^19^, ease of printing on flexible substrates^23^ etc. Numerous demonstrations of electrolyte gated CNTFETs have been reported where the dielectric is either an electrolyte in aqueous form^24^ or is a solid or ionic-gel electrolyte^25^. Applications seeking low applied voltages like bio-sensing which can be integrated to wearable devices, or thin film batteries^26^ have been the major impetus for the growing research area of EG-CNTFETs. Towards this aim, capability to fabricate electronic circuits using EGFETs, which in turn call for the capability to produce efficient n-type CNTFETs, is a requirement.

In this work we demonstrate for the first time an aqueous gated n-type CNTFET, which is solution processable and is fabricated by relatively simple and cost-effective techniques (requiring no high-temperature processes). The n-type behavior is obtained by simple modification of the channel of the as fabricated classical p-type CNTFET. An additional tailored modifying layer stabilizes negative charges on CNTs that occur when a positive bias voltage is applied on the gate. The channel is modified by drop casting dispersion of a polymer containing composition of lipophilic and mobile ions, followed by spontaneous ambient atmosphere drying that results in formation of a membrane layer on top of the channel. The composition of this poly(vinyl chloride) (PVC) based layer is tailored to enable ionic-conductivity in the lipophilic phase, yet also preventing significant ion exchange with solution, benefiting from properties of the ion-exchangers used. The lipophilic ions (anions and cations) present in the membrane layer are mobile within the phase but due to high lipophilicity are unlikely to be transferred to the aqueous phase of the electrolyte^27^. Nevertheless, upon polarization charge separation within this layer can be induced (with preservation of overall neutrality). Additionally, the membrane layer contains relatively small fraction of mobile ions that upon extensive polarization can be reversibly expelled from the phase to the adjacent solution, ultimately resulting in momentary partial positive charge on the membrane layer and allowing n-doping of the CNTs underneath. Upon polarization change these ions are re-entering the membrane layer allowing the whole system to achieve electroneutrality.

## Methods

**Figure 1:**
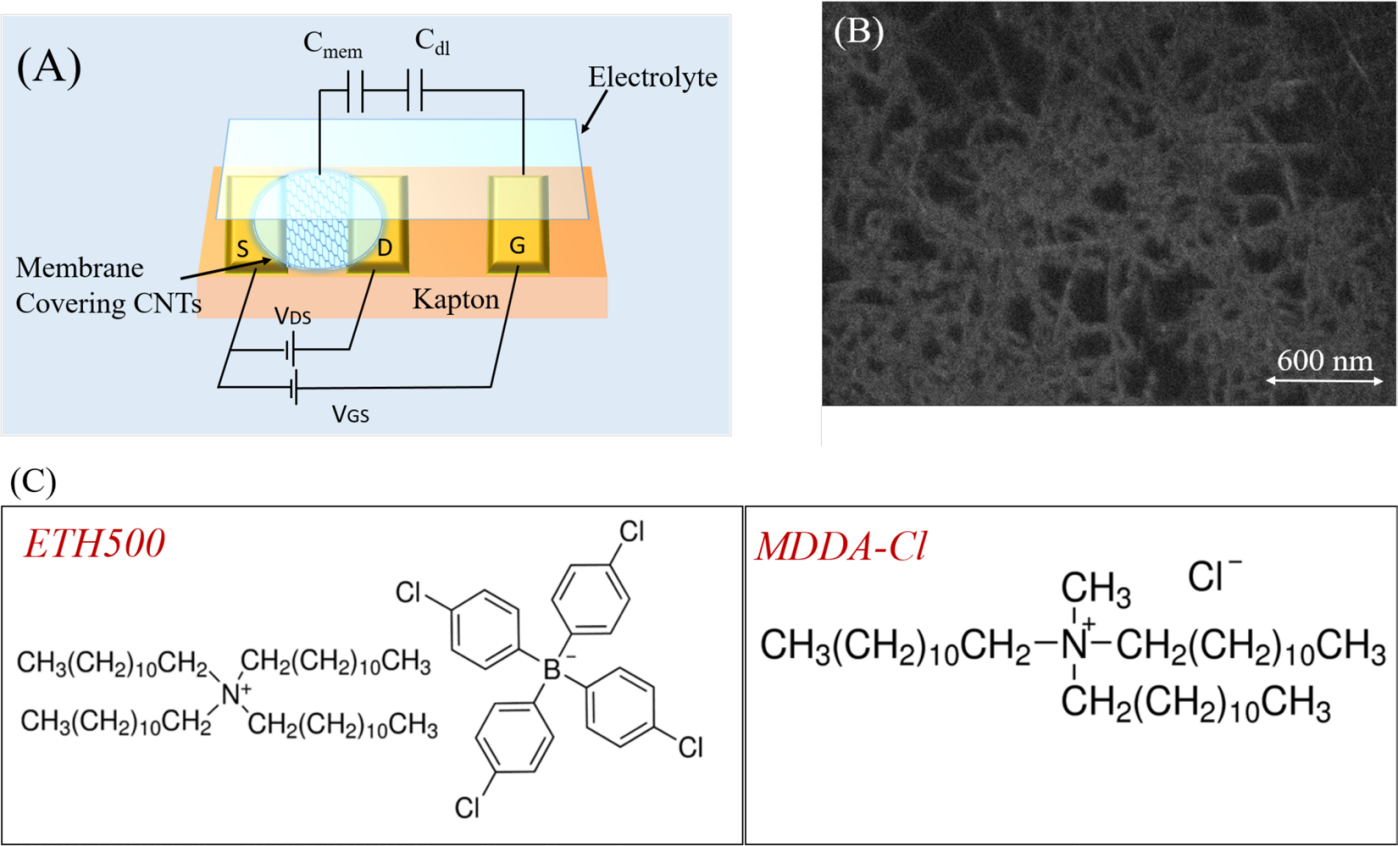
(**A**) Schematic of CNTFET, (**B**) SEM of the CNT channel. (**C**) Chemical structure of tetradodecylammonium tetrakis(4-chlorophenyl)borate (ETH 500) and methyltridodecylammonium chloride (MDDA-Cl).

The CNTFETs are fabricated on a flexible polyimide substrate (Kapton ©, DuPont, 300 HN, 150 *μ*m). Interdigitated source-drain and gate electrodes are formed by an adhesion promoter layer of 5 nm chromium with 50 nm gold layer, which are patterned via standard photo-lithography process. The active channel area of the transistor, with the channel length of 75 *μ*m and aspect ratio of 440, is formed by a random network of semiconducting CNTs (90% purity, Sigma-Aldrich) of mean diameter 0.77 nm and average length 770 nm. The CNTs are dispersed in aqueous medium surfactant sodium dodecyl sulfate (SDS) and sprayed through a shadow mask^28^ using an automated spray system equipped with industrial air atomizing spray valve (Nordson EFD) and an overhead motion platform (Precision Valve Automation)^29^. Post spray, the samples are immersed in DI-H_2_O to remove surfactant from the CNT network. Figure 1(A) shows the schematic of the electrolyte gated (EG)-CNTFET and 1(B) is the SEM image of the sparse carbon nanotube (CNT) network. The SEM image confirms the randomness of the CNT network indicating that the percolation path of the current is probabilistic in nature and hence there will be variations in the transistor characteristics from one device to another^23^.

The active channel of the CNTFET is covered with a tailor lipophilic membrane layer. This membrane layer contains 31.3% poly-vinyl chloride (PVC) which provides the structural support to the membrane^30^ and 63.6% dioctyl sebacate (DOS) which acts as a plasticizer^31^. The lipophilic ion additives of this layer are 3% tetradodecylammonium tetrakis(4-chlorophenyl)borate (ETH 500) and 2.1% methyltridodecylammonium chloride (MDDA - Cl). Thus, the membrane layer contains cations and anions of mobility constrained to the lipophilic phase, tetradodecylammonium and tetrakis(4-chlorophenyl)borate respectively, as well as some anions (Cl^−^) free to be exchanged through the membrane layer-solution interface. The final membrane layer solutions are prepared by dissolving these constituents completely in 1 ml of THF (tetrahydrofuran), and 10 μl of this solution is dropcast over the entire CNTFET channel and the devices are left to dry in ambient atmosphere. Simple fabrication steps including dropcasting of membrane layer makes the entire process easily scalable and automatized. The chemical structure of ETH500 and MDDA - Cl are shown in Fig. 1(C).

To understand the mechanism of ion exchange giving rise to the n-type behavior in CNTFETs, a fluorescent dye (RBOE (Rhodamine B octadecyl ester perchlorate)) is introduced additionally in the lipophilic salt and the emission spectra are studied. This membrane composition is conditioned in the solution containing 2.4 mg of the RBOE in 1 ml of ethanol and DI-H_2_O (1:1 by volume), to achieve surface positioning of the dye (due to spontaneous absorption) in the open circuit initial condition.

## Results and Discussion

**Figure 2:**
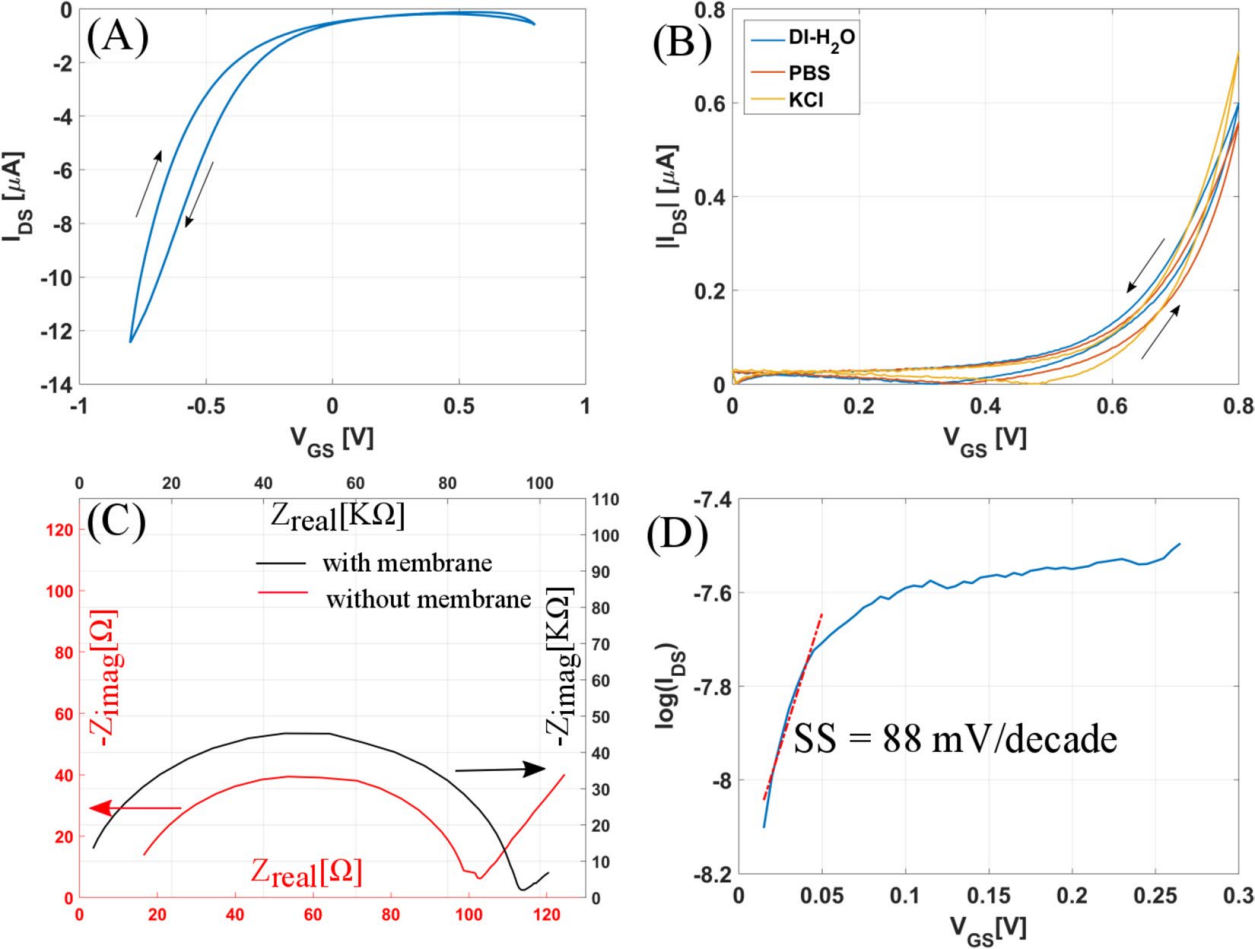
Channel covered with lipophilic membrane layer: Transfer Curve (I_*DS*_ vs V_*GS*_) (**A**) before and (**B**) after the channel is encapsulated. (**C**) Impedance spectroscopy results: imaginary part (Z_*imag*_) vs real part of impedance (Z_*real*_) before and after a gold electrode with CNTs is encapsulated by the membrane. (**D**) Sub logarithmic plot of current vs gate voltage showing the subthreshold swing of the n-type CNTFET.

Figure 2(A) shows the transfer curve of the bare as-fabricated CNTFET with 20 mM PBS (phosphate buffer saline) as the electrolyte. A classical p-type response of the CNTFET is observed, the transistor turns on as the gate to source voltage (V_*GS*_) is swept from +0.8 V to −0.8 V and the drain to source voltage (V_*DS*_) is fixed to −0.2 V. Application of a membrane on the channel results in pronounced change in the device characteristics as seen in Fig. 2(B). The orange curve in Fig. 2(B) shows the transfer curve with 20 mM PBS as gate electrolyte depicting a n-type response, with the applied V_*GS*_ sweeping from 0 to +0.8 V and V_*DS*_ fixed to −0.2 V. Qualitatively the same behavior is observed in other electrolytes tested, namely DI-H_2_O and saturated KCl. The drain to source current (I_*DS*_) is maximum when the gate electrolyte is KCl. This is because an increasing amount of Cl^−^ anions in the electrolyte solutions leads to easy exchange of Cl^−^ between the membrane layer and solution. The arrows in Fig. 2(A) and (B) indicate the direction of voltage applied and give an idea about the hysteresis in the CNTFETs. Hysteresis is an electrical instability mostly attributed to oxygen and water molecules adsorbed on the CNT surface or any interface or surface traps^32,33^.

However, there is overall decrease in I_*DS*_ after encapsulation of the channel with the membrane layer. There are two reasons for this behavior. One is the decreased effective capacitance between the gate and channel of the CNTFET, because of the introduction of an additional membrane capacitance (C_mem_). In an electrolyte gated FET, the gate capacitance is defined by the two double layer capacitances (C_dl_) formed respectively at the gate-electrolyte interface and the electrolyte-semiconductor interface^19^. However, after introduction of the membrane layer an additional small capacitance is introduced in series with the double layer capacitance. Hence the effective capacitance defined as C_eff_ is lower than C_*dl*_^19^.

$$\frac{1}{{C}_{{\rm{eff}}}}=\frac{1}{{C}_{{\rm{dl}}}}+\frac{1}{{C}_{{\rm{mem}}}}$$

Figure 2(C) shows the complex plane impedance plots for electrode impedance spectroscopy measurements, carried out in 0.1 M KCl solutions with glassy carbon working electrode coated by a carbon nanotube layer with and without membrane, using an amplitude of 50 mV at a potential of 0.5 V. The capacitance is calculated using the relation *ω*RC = 1 at the point of maximum Z_*imag*_. There is three orders of magnitude decrease in the capacitance after the membrane layer encapsulation. Another factor that contributes to the decrease in current is the increase in the resistance between source and drain, which is two orders of magnitude in this case. (Also, refer to supporting information Figure S1). However, the lowering of drain to source current does not essentially impacts the on-off ratio of the CNTFET, as the on-off ratio of the modified n-type CNTFET is almost twice of the bare p-type CNTFET. Semilog plots for the p-type and n-type CNTFET are shown in Supporting information Figure S2. Comparing the drain to source current values to two recent works^16,17^ where p-type CNTFETs are converted to n-type, the applied voltages needed are in tens of volts to achieve currents in *μ*A range and the transistors are also not water stable.

The parameters for the n-type transistor shown in Fig. 2(B) are calculated using the data obtained in PBS electrolyte. The on-off ratio is 200 and the maximum transconductance (g_*m*,*max*_) is 0.1 *μ*S. The threshold voltage (V_*th*_) calculated using the ELR (extrapolation in the linear region) method is 0.25 V. The subthreshold swing (SS) is 88 mV/decade, calculated as shown in Fig. 2(D) which is the semi-logarithmic plot of I_*DS*_ vs V_*GS*_ plotted for voltages from 0 to V_*th*_. Table 1 presents the statistical data for key parameters like current on-off ratio, threshold voltage, hysteresis and maximum transconductance for six CNTFETs before and after the channel is encapsulated with the membrane.

**Table 1:** Comparison of the major device characteristics like on-off ratio, threshold voltage, hysteresis and transconductance for the p-type CNTFET and after it is encapsulated by the membrane to form n type CNTFET.

	on-off ratio	V_*th*_	V_*HY ST*_	(g_*m*_,_*max*_)
p-type (Without Membrane)	85 ± 33	−0.53 ± 0.2 V	0.06 ± 0.04 V	41 ± 20 *μ*S
n-type (With Membrane)	222 ± 74.04	0.36 ± 0.25 V	0.025 ± 0.015 V	0.1 ± 0.037 *μ*S

**Figure 3:**
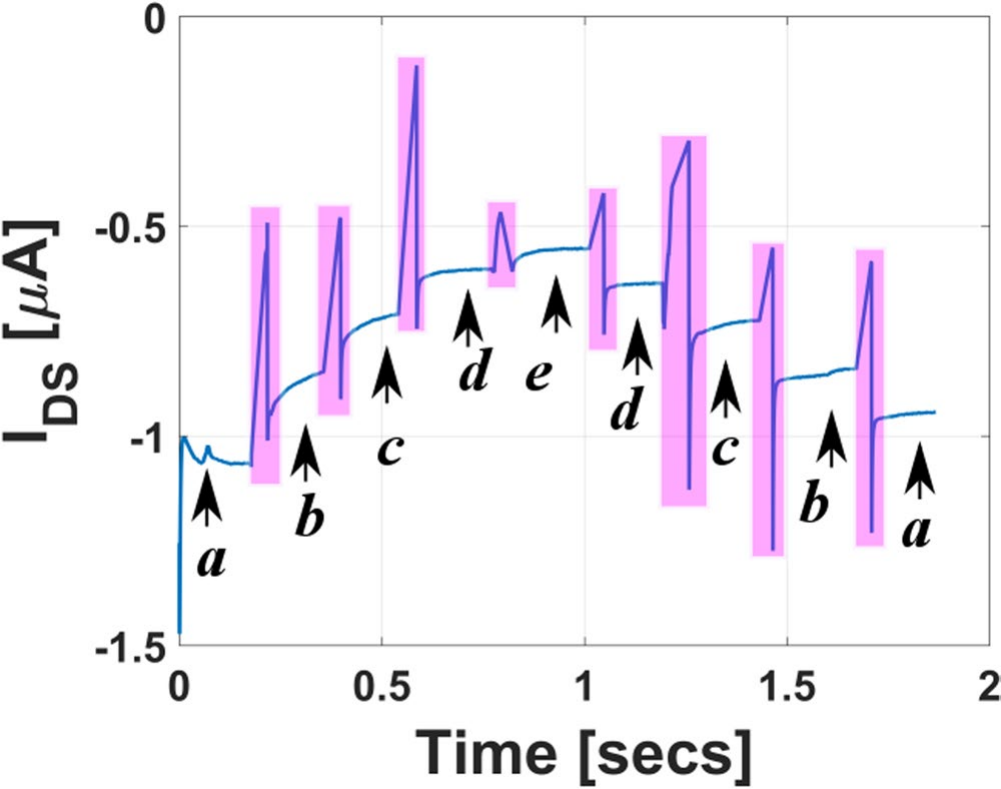
pH response of the modified CNTFET, (pH values indicated in the figure a = 7.6, b = 5.5, c = 4.5, d = 3, e = 2). The magenta color indicates the period of solution exchange.

To establish the potential use of such n-type modified FETs in sensing applications, the pH response of the modified n-type CNTFET is recorded. The device is challenged to pH solutions varying from 7.5 to 2, and fixed voltage biases are applied at the three terminals such that V_*GS*_ = 0.8 V and V_*DS*_ = −0.2 V. The pH response shown in Fig. 3 demonstrates that the drain current decreases for the n-type CNTFET with decreasing pH. This response is as expected and opposite to that seen for a p-type CNTFET where the current increases with decreasing pH^28^. A decrease in pH leads to an increase in the concentration of H^+^ which is accompanied by increase in the anion concentration (coming from the acid used to acidify the solution) on the expense of decrease in OH^−^ concentration. Increased anion concentration around the membrane impedes the chloride ion exchange across the membrane-solution interface and hence the decrease in current.

The mechanism of the transition from a p-type behavior to n-type of the CNTFET is attributed to the charge separation in the lipophilic phase on negative polarization of the membrane layer. When a positive bias is applied on the gate, the membrane layer is negatively polarized and hence the mobile chloride ions are expelled from the PVC matrix to the solution. This results in the *temporary* positive charging of the membrane layer due to extra cations present in the phase, which is stabilized by negative charges on the random CNT network located underneath. As a result the n-type behavior is seen. It can be assumed that this process is accompanied by the movement of lipophilic cations in the membrane closer to the CNT network.

**Figure 4:**
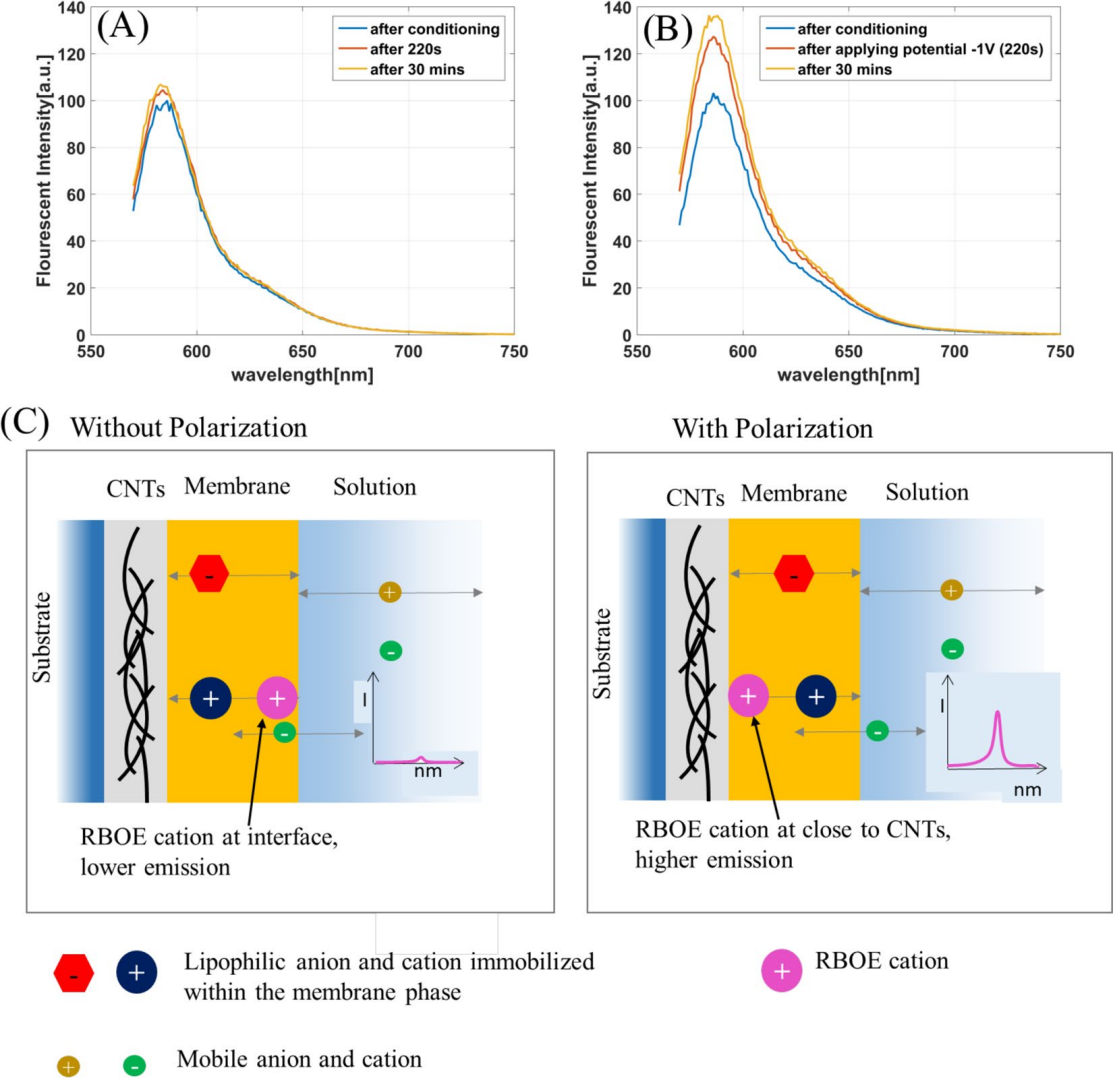
(**A**) Control experiment, the emission spectra are recorded without any electrochemical trigger. (**B**) A negative polarization of −1V is applied on the membrane after 220 secs. (**C**) Schematic showing the ionic movement in the absence and presence of polarization.

To prove the above postulated mechanism a model experiment is performed using a membrane of similar composition, with the difference that the lipophilic salt (RBOE-ClO_4_) is characterized additionally with the ability of the cation used to emit fluorescence of different intensity depending on the environment. Movement of the RBOE cation from the membrane-solution interface towards the CNT-membrane interface is expected to result in an increase of emission intensity due to an increase in the lipophilicity of the environment^34^. These experiments are performed on glassy carbon electrodes, conditioned in KCl solution to mimic conditions of n-type transistor operation. Figure 4(A) shows a control experiment where no polarization is applied on the membrane. The emission spectrum is recorded three times, once immediately after conditioning, once after 220 secs and then after 30 mins. There is no difference in the fluorescent intensity in the three cases. However, when a negative polarization is applied on the membrane as in Fig. 4(B), there is significant increase in the intensity for the RBOE cation in comparison to that recorded before the electrochemical trigger. This suggests that the RBOE cation (after polarization) is in a more lipophilic environment. This is a clear indication that polarization affects ionic movement including that of a fluorescent cation in the membrane, from the outer interface towards the more lipophilic bulk, coupled with expulsion of mobile anions from the phase. The schematic representation of the ionic movement upon polarization is shown in Fig. 4(C). This effect is however reversible and upon disconnecting the system, i.e. in the absence of polarization – a decrease in emission is observed. Thus, pointing to movement of fluorescent cation towards a more hydrophilic environment, i.e. membrane-solution interface.

**Figure 5:**
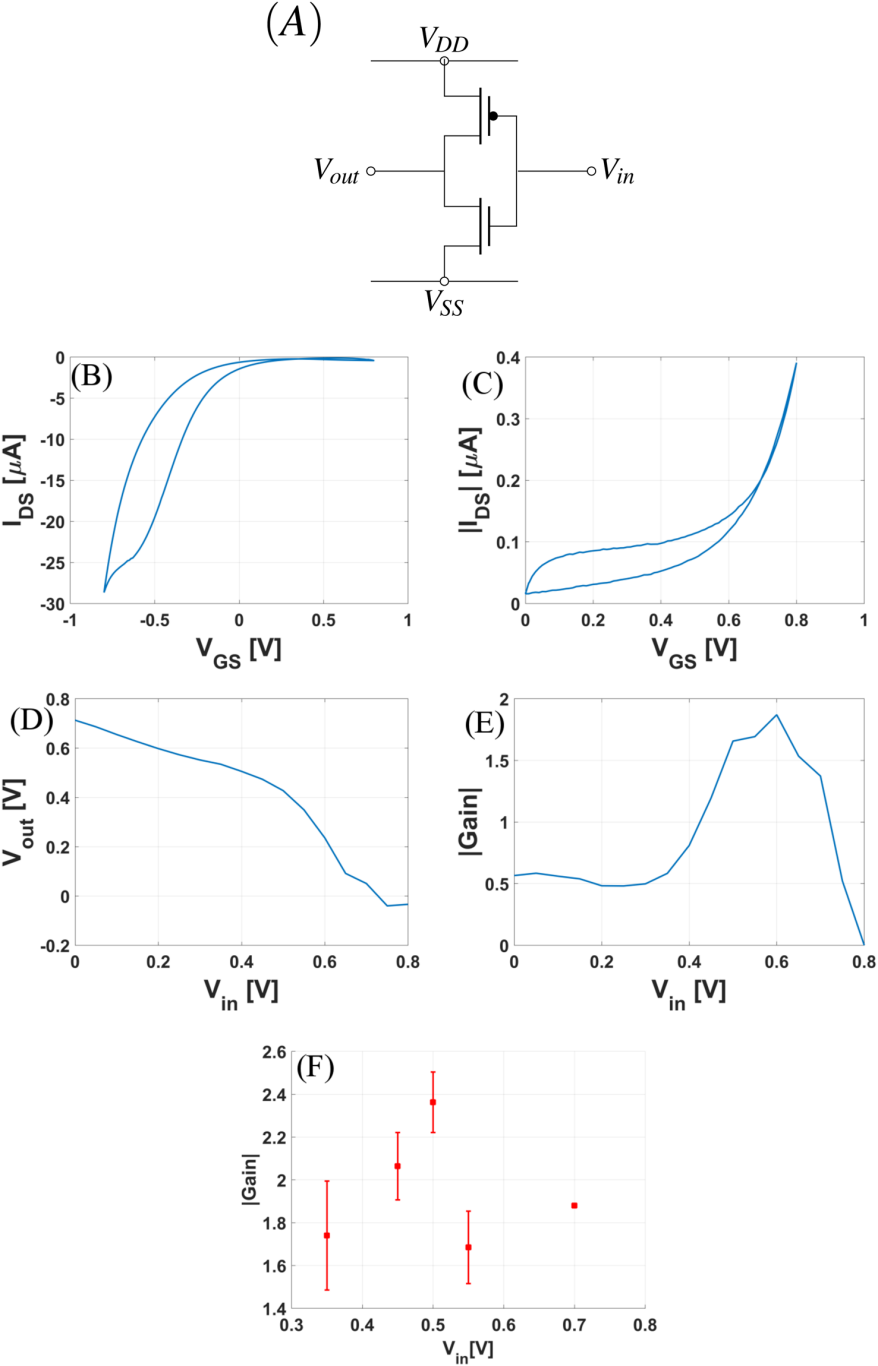
(**A**) Schematic of a CMOS inverter. Transfer curve for the (**B**) p-type and (**C**) n-type CNTFETs of the inverter. Input-output (I-O) characteristic for the inverter. (**D**) V_*out*_ vs V_*in*_ (**E**) Gain vs V_*in*_. (**F**) Gain vs V_*in*_ for five different inverters.

Finally, a p-type CNTFET and a membrane modified n-type CNTFET are used to fabricate a CMOS electrolyte gated inverter, in the configuration shown in Fig. 5(A). Figure 5(B) and (C) show the transfer characteristics for the p-type and n-type FETs of the inverter measured in 20 mM PBS buffer. To characterize the CMOS inverter, the input voltage (V_*in*_) which is the bias applied at the gate of the two FETs is swept from 0 to 0.8 V and the two supply biases are fixed as V_*DD*_ = 0.8 V and V_*SS*_ = −0.8 V. Figure 5(D) and (E) show the input-output characteristic of the inverter. The maximum gain of this inverter is close to 2. Although the aqueous electrolyte provides the opportunity of working in extremely low (sub 1 V biases), it also provides a challenge that in a very small input voltage range we do not obtain a sharp noise margin. However, in applications where a higher noise margin is a stringent requirement ionic gels^35,36^ can be used and better performance can be achieved by applying higher voltages (beyond 1 V). Figure 5(F) shows the gain vs input voltage plot for such five fabricated inverters.

## Conclusion

In this work we have for the first time demonstrated a aqueous gated n-type CNTFET. Lipophilic membranes modified by the addition of ion-exchangers encapsulate the CNT channel, causing ion exchange at the membrane electrolyte interface. Lipophilic cation movement within the membrane phase renders an n-type behavior to the CNTFET. Such transistors can be used for sensing applications as demonstrated by pH sensing measurements. Additionally, aqueous gated CMOS inverter for the very first time have been demonstrated using modified p- and n-type CNTFETs operating in applied voltages below 1 V.

## Electronic supplementary material

Supplementary File
