# Supplementary material for: Ambient Processed, Water-Stable, Aqueous-Gated sub 1 V n-type Carbon Nanotube Field Effect Transistor
Source: Sci Rep. 2018 Jul 30;8:11386. doi: 10.1038/s41598-018-29882-w (PMC6065383; doi:10.1038/s41598-018-29882-w)

Supporting Information

Ambient Processed, Water-Stable, Aqueous-Gated sub 1V n-type Carbon Nanotube Field Effect Transistor

Saumya Joshi[1,*], **Vijay Deep Bhatt**[1], **Ewa Jaworska**[2], **Agata Michalska**[2,+,*], **Krzysztof Maksymiuk**[2,+], **Markus Becherer**[1], **Alessio Gagliardi**[1], **and Paolo Lugli**[3,+]

[1]Department of Electrical and Computer Engineering, Technische Universit ¨ at M¨unchen, Munich 80333, Germany.
[2]Faculty of Chemistry, University of Warsaw, 02093 Warsaw, Poland
[3]Faculty of Science and Technology, Free University of Bozen-Bolzano, 39100 Bolzano, Italy
[*]saumya.joshi@tum.de
[*]agatam@chem.uw.edu.pl
[+]these authors contributed equally to this work

Figure S1:- (A) Resistance information before and after membrane. There is cconsiderable decrease in the source-drain current before and after the channel of the CNTFET is encapsulated with membrane. For these measurement the gate electrode is floating and the resistance is measured in absence of electrolyte. (B) Zoom in view of the current-voltage after membrane is applied on the channel

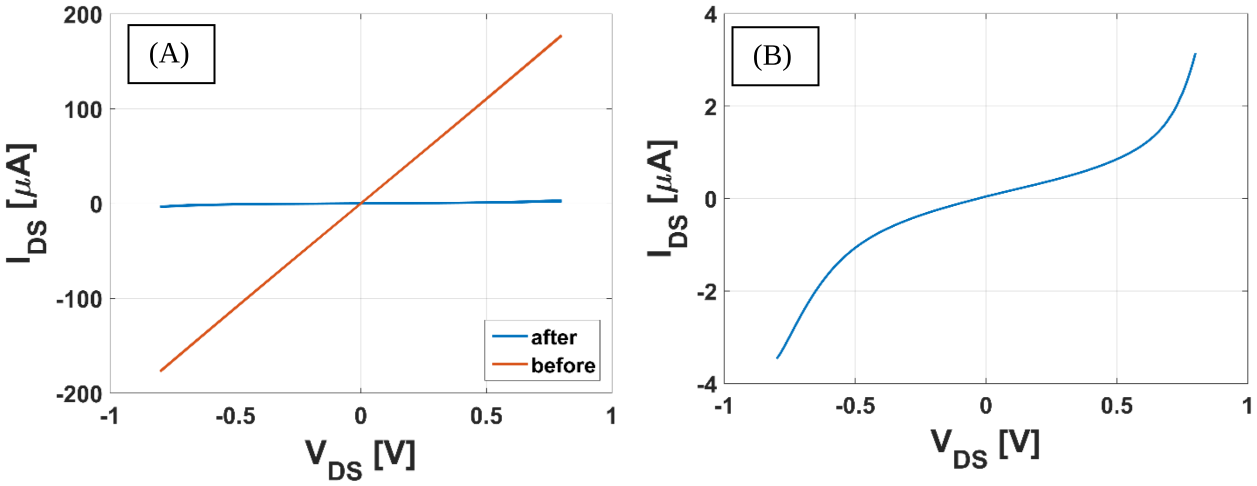

Figure S2:- Semilog plot for the CNTFET shown in Figure 2, (A) before and (B) after CNT channel is encapsulated by the membrane.

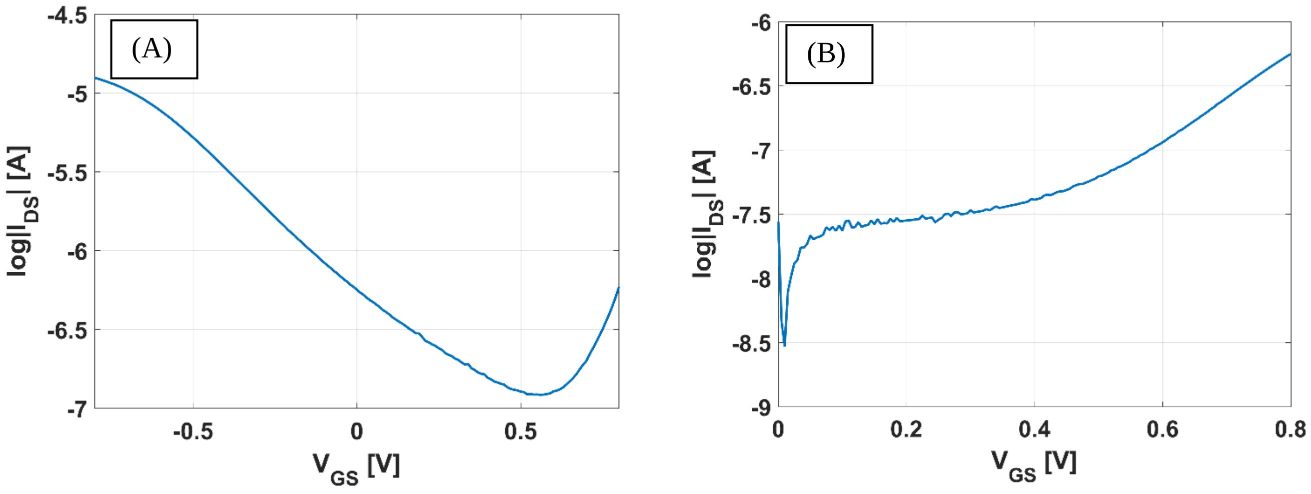

Figure S3:- Transfer curve for a modified n-type CNTFET when the applied gate volatge in the range from -0.8 to +0.8V. The blue line indicates first measurement cycle and orange is the second measurement cycle.

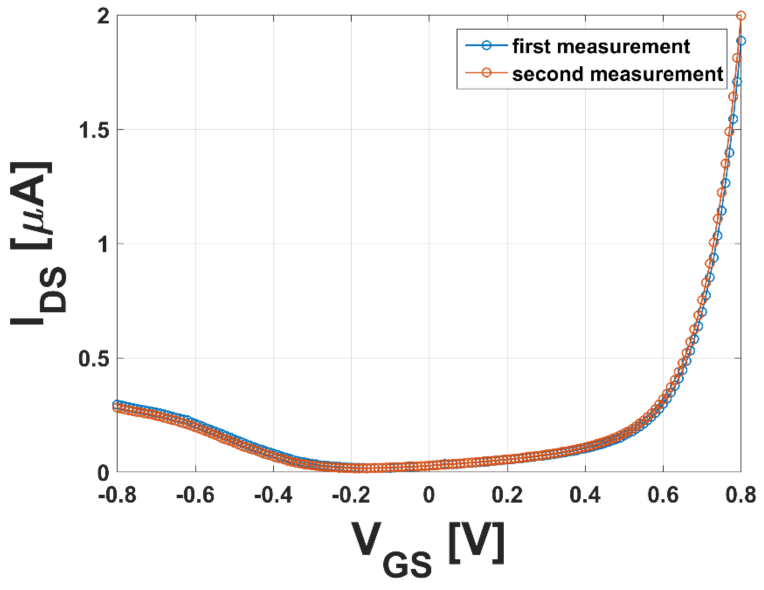

Figure S4:- Transfer curve for the n-type CNTFET measured in saturated KCl after fabrication and re-measured again after two days to get an idea about the stability of the device.

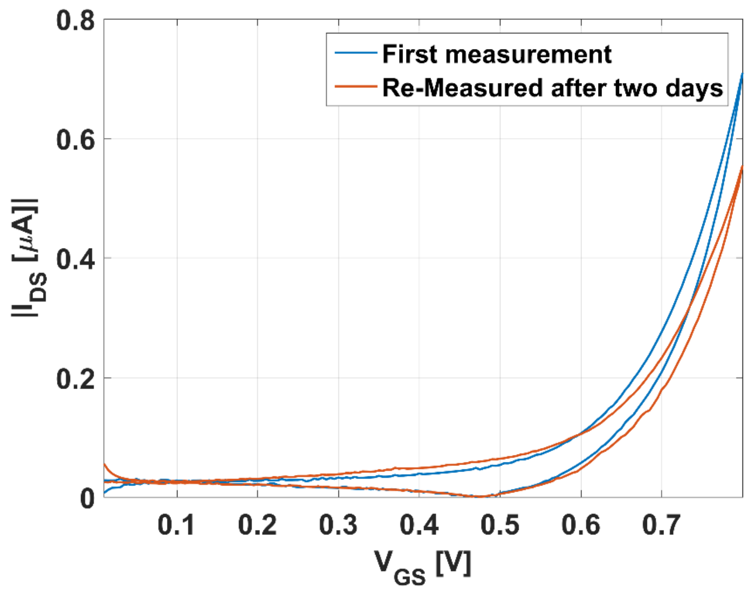

Figure S5:- Transfer curve for p-type CNTFET measured in PBS, KCl and DI-H2O.

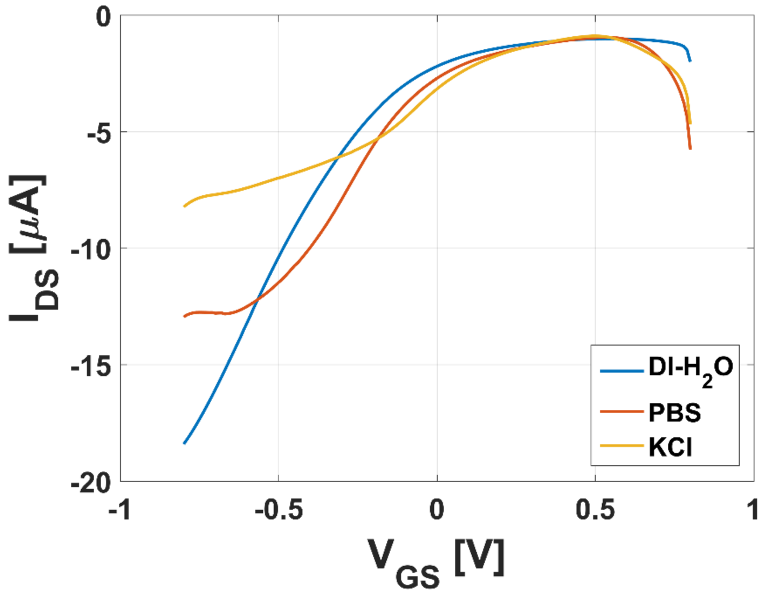

Supplement: Supplementary file 1 — Supplementary File [file 41598_2018_29882_MOESM1_ESM.pdf]
